# First person – Panagiota Giardoglou and Despina Bournele

**DOI:** 10.1242/bio.058665

**Published:** 2021-03-11

## Abstract

First Person is a series of interviews with the first authors of a selection of papers published in Biology Open, helping early-career researchers promote themselves alongside their papers. Panagiota Giardoglou and Despina Bournele are co-first authors on ‘A zebrafish forward genetic screen identifies an indispensable threonine residue in the kinase domain of PRKD2’, published in BiO. Panagiota is a PhD student in the lab of Dr Dimitris Beis at the Biomedical Research Foundation Academy of Athens, Greece, investigating modelling human cardiovascular diseases and underlying the mechanisms involved in their pathophysiology. Despina is a toxicologist in the lab of Dr Kyriaki Machera at the Benaki Phytopathological Institute, Kifissia, Athens, Greece, investigating molecular and developmental biology, and zebrafish toxicology.


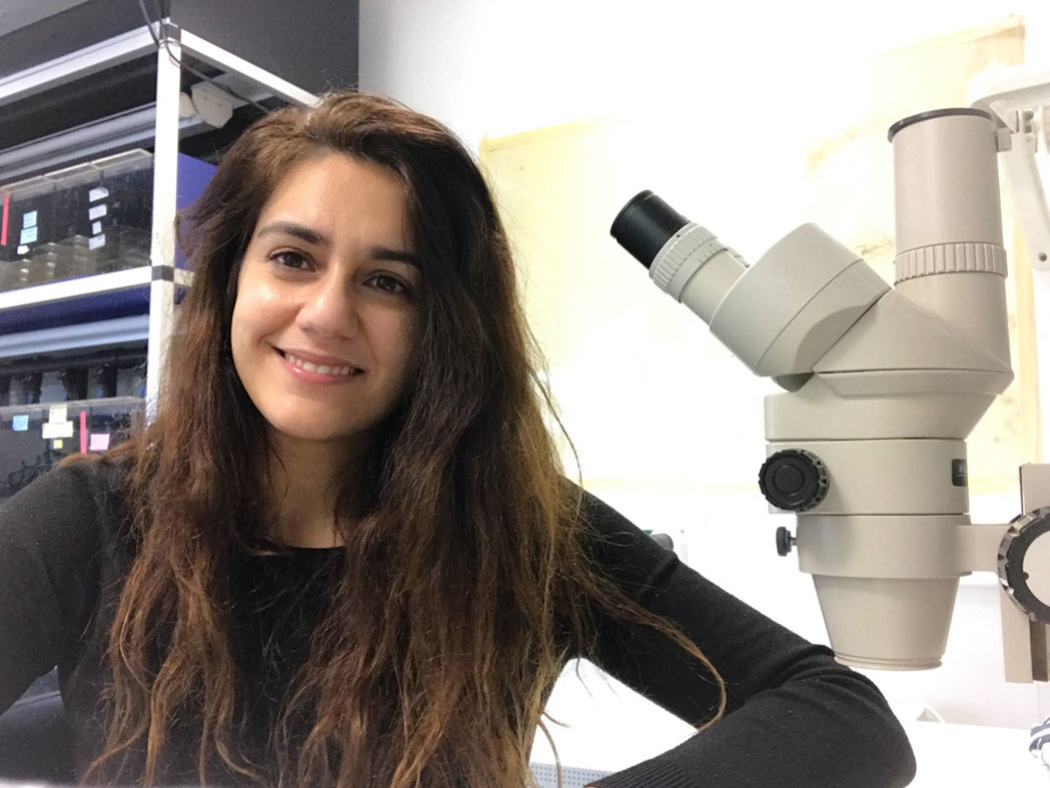


**Panagiota Giardoglou**


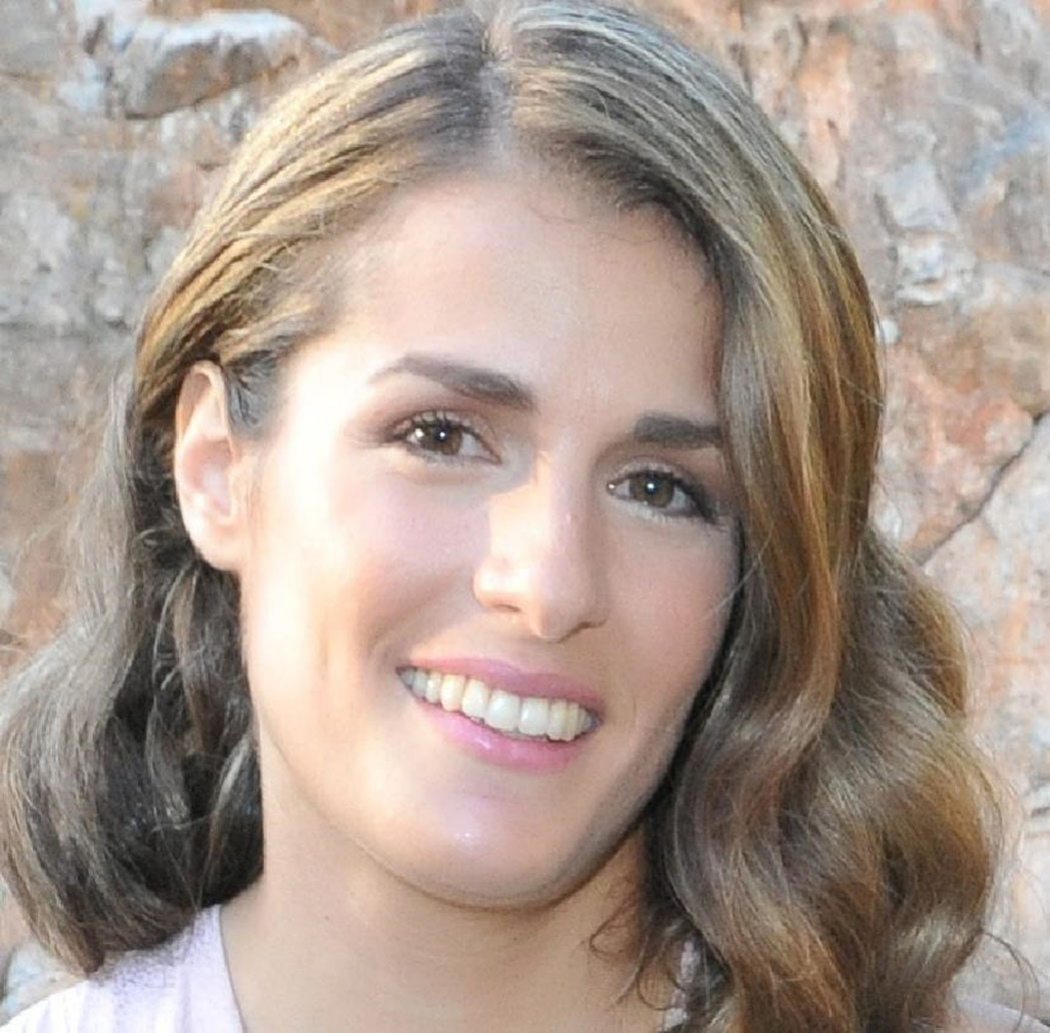


**Despina Bournele**

**What is your scientific background and the general focus of your lab?**

**P.G.:** I am a biologist and along my scientific journey, I had the opportunity to work with a variety of research models (rodents, zebrafish, plants) in the fields of developmental and cellular biology. Through that, I was fascinated by how strong research findings in small animals could lead to extrapolation in human conditions/diseases and their possible therapeutic strategies. Hence, my inclination towards the modelling of specific human cardiac disorders in zebrafish and the dissection of causal interactions resulting in the appearance of disease traits led me to start a multidisciplinary PhD program and join Dr Dimitris Beis's lab at BRFAA and Professor Dedoussis at HUA University. The main focus of the Beis lab is to elucidate the signalling pathways that orchestrate the morphogenesis and function of heart development *in vivo* and study complex processes such as cardiac valve formation using forward and reverse genetic approaches as well as state-of-the-art techniques and experimental tools.

**D.B.:** My academic training is in biology and my research background focuses on molecular and developmental biology. My PhD thesis was in zebrafish models of cardiovascular diseases, where under the mentorship of Dr Dimitris Beis I studied a zebrafish cardiovascular mutant with an outflow tract stenosis phenotype. During my PhD thesis, I was granted a scholarship from the PhD Scholarship Programme ‘Heraclitus II’.

**How would you explain the main findings of your paper to non-scientific family and friends?**

Since cardiac disorders during development are lethal in most organisms, it is very crucial to discover the master regulators that are involved. In our paper, we utilize zebrafish, a valuable animal model used to address significant questions about factors implicated in the pathogenesis of congenital heart diseases in humans. Zebrafish embryos named ‘bulbulithra’ develop a complete valve stenosis that blocks the blood between ventricle and atrium. As a result, they die at a late embryonic stage but still offer a time window where we can study aspects of developmental abnormalities. We discovered that the gene responsible for this cardiac extreme defect is *pkrd2*, which is also involved in many human heart disorders and we linked it to both known and novel major signalling pathways. Hence, we created a valuable platform to further study the heart valve development as well as a tool to test candidate targets for therapeutic purposes.

**What are the potential implications of these results for your field of research?**

Our paper accounts for another valuable arrow in the quiver of the researchers in the heart development and human disease model field. Here, we highlight the power of forward genetics, we identify a specific critical residue of *prkd2* that causes a complete inactivation of the PRKD2 kinase and we reveal a novel link of its function to pathways such as calcineurin and TBX5 that was not shown before. This provides not only a residue target for *prkd2* pharmaceutical inactivation but also an important tool-model for screening therapeutic candidates for valve malformations.

“Our paper accounts for another valuable arrow in the quiver of the researchers in the heart development and human disease model field.”

**What has surprised you the most while conducting your research?**

In terms of science, it's extremely exciting that a small fish carries such a high similarity to human in the levels of genomes, proteins and physiology (albeit mankind doesn't exactly develop the same swimming skills). Based on that, it was surprising that a highly conserved gene when mutated develops so extreme a cardiac phenotype in zebrafish and yet, despite that fact, we could still use the biological advantage of the model and characterize further the phenotype and the involved pathways. Also, our work was initiated a long time ago and it required years until it was completed. Thus, this is a good example of how persistent you have to be in research in order to finally achieve your goals.

**Figure:**
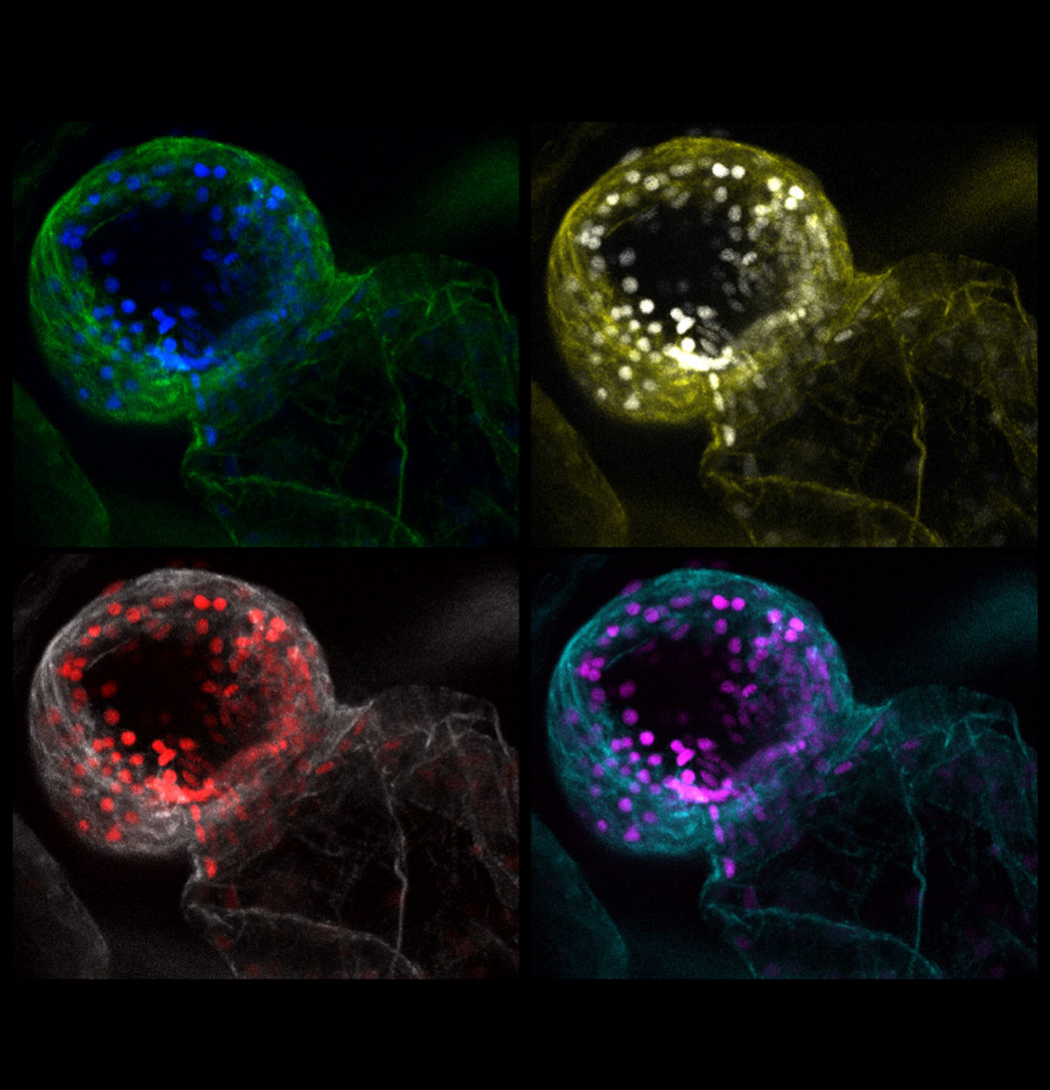
**Confocal image of embryonic cardiac slice from *s411* mutant zebrafish *Danio rerio* depicting the Notch-activated cells that ectopically reside throughout the ventricular endocardium. Expression of Notch pathway is visualized by *Tg(TP1:mcherry)* reporter while the myocardium is stained with 633-phalloidin.**

**What, in your opinion, are some of the greatest achievements in your field and how has this influenced your research?**

**P.G.:** As part of my PhD, I work in two different projects both utilizing zebrafish as a model system and its great advantages in the fields of heart development and modelling of human diseases. In the last few decades, genome editing tools have been developed and have changed the whole prospective dynamic of life sciences. The flagship of these tools is the molecular fine-scissors of Crispr/Cas9 system that has been a revolution leading to a real burst of discoveries. This milestone achievement and its greater extensions gave scientists the opportunity to create *in vivo* and *in vitro* models to study the role of a gene, or even of a single nucleotide variant. After optimization and application of this system also in our lab, I generated a knockout zebrafish line targeting a gene that based on GWAS is involved in the appearance of cardiovascular disease and the results of this study will hopefully be published in our next research paper.

**D.B.:** Although zebrafish has been used as an animal model since the 1940s, researchers are taking full advantage of and understanding its potential only the last few years. Zebrafish exhibits a unique set of properties and is increasingly used worldwide as a model organism in biomedical research for answering fundamental and applied problems in biological and health-related fields.

**What changes do you think could improve the professional lives of early-career scientists?**

**P.G.:** Interaction, networking, knowledge sharing and collaborations are precious key points that a scientist must build and constantly work on throughout their career. As new technologies emerge and discoveries on the same subject in different places of this world rise, it is important that we invest in fruitful scientific interconnection so that we overcome communication barriers, move our research forward and promote the ultimate aim of science: to understand, predict, and explain.

**D.B.:** The most important problem that early-career scientists face is the limited research funding. This often leads to many scientists ending their academic careers and choosing permanent and well-paid positions in industry or elsewhere. Increased research funding sources would significantly improve the professional lives of early-career scientists.

**What's next for you?**

**P.G.:** Definitely my PhD defence, that's coming up first. After completing my doctorate, I would love to continue as a postdoctoral researcher using my favourite experimental model, *Danio rerio*, and work on critical questions emerging from ‘the matters of the heart’.

**D.B.:** Since completing my PhD thesis, I have been working in toxicology at Benaki Phytopathological Institute. I believe that zebrafish is an ideal vertebrate model for high-throughput drug testing and I would like to continue my research career in the field of zebrafish toxicology.
